# Transcriptome Analysis Reveals Key Genes Involved in Weevil Resistance in the Hexaploid Sweetpotato

**DOI:** 10.3390/plants10081535

**Published:** 2021-07-27

**Authors:** Kanoko Nokihara, Yoshihiro Okada, Shinichiro Ohata, Yuki Monden

**Affiliations:** 1 Graduate School of Environmental and Life Science, Okayama University, Okayama, Okayama 700-8530, Japan; p97j1mjy@s.okayama-u.ac.jp (K.N.); ph563a4t@s.okayama-u.ac.jp (S.O.); 2 Kyushu Okinawa Agricultural Research Center, National Agriculture and Food Research Organization, Itoman, Okinawa 901-0336, Japan; yoc1973@affrc.go.jp

**Keywords:** transcriptome, RNA-seq, sweetpotato, weevil resistance, juvenile hormones, terpenes

## Abstract

Because weevils are the most damaging pests of sweetpotato, the development of cultivars resistant to weevil species is considered the most important aspect in sweetpotato breeding. However, the genes and the underlying molecular mechanisms related to weevil resistance are yet to be elucidated. In this study, we performed an RNA sequencing-based transcriptome analysis using the resistant Kyushu No. 166 (K166) and susceptible Tamayutaka cultivars. The weevil resistance test showed a significant difference between the two cultivars at 30 days after the inoculation, specifically in the weevil growth stage and the suppressed weevil pupation that was only observed in K166. Differential expression and gene ontology analyses revealed that the genes upregulated after inoculation in K166 were related to phosphorylation, metabolic, and cellular processes. Because the weevil resistance was considered to be related to the suppression of larval pupation, we investigated the juvenile hormone (JH)-related genes involved in the inhibition of insect metamorphosis. We found that the expression of some terpenoid-related genes, which are classified as plant-derived JHs, was significantly increased in K166. This is the first study involving a comprehensive gene expression analysis that provides new insights about the genes and mechanisms associated with weevil resistance in sweetpotato.

## 1. Introduction

Sweetpotato (*Ipomoea batatas* (L.) Lam.) is a member of the Convolvulaceae family that is widely cultivated in the tropical and temperate zones. As a valuable source of carbohydrates, vitamins, fiber, and minerals, sweetpotato is considered one of the most important crops in the world, with an annual production of over 100 million tons globally [1]. In recent years, the production of sweetpotato varieties with favorable cultivation-related traits, such as high added-value and resistance to diseases, pests, and low soil temperature, was conducted for the expansion of planting areas. However, sweetpotato is a typical non-model crop species and a hexaploid (2n = 6x = 90) with a complex mode of inheritance and a large genome (2–3 Gb); hence, breeding and genetic studies have been difficult. Furthermore, although some varieties can be self-fertile, most show self-incompatibility or mating incompatibility with a specific group of varieties. This reproductive pattern causes the sweetpotato genome to be highly heterozygous. Therefore, it is extremely difficult to identify the genes and the underlying molecular mechanisms associated with important agricultural traits in sweetpotato.

Two weevil species, namely *Cylas formicarius* (Fabricius) and West Indian sweetpotato weevil (*Euscepes postfasciatus* (Fairmaire)), are known as the most damaging pests of sweetpotato worldwide [2,3], causing economic damage to farmers in Central and South America and the South Pacific Islands [4]. In Japan, weevils are distributed in tropical and subtropical regions, including the Nansei and Ogasawara Islands [5]. Particularly in Okinawa, sweetpotato is an essential crop that supports the backbone of its tourism industry. However, the crop yield is 60% less than that of mainland Japan; this low yield is mainly due to the feeding damage caused by weevils. Therefore, the development of weevil-resistant cultivars is important to mitigate this problem. Both species can invade the root approximately 2 months after planting and then lay eggs. The hatched larvae move to the tuberous roots while feeding on the inside of the stem and eventually become pupae that hatch into adults, which break through the tuberous roots and escape to the outside, becoming a new generation of parent insects. Therefore, weevils spend most of their lifetime either in stems or roots, shielding them from insecticides and causing significant damage to sweetpotato plants. The tuberous roots damaged by feeding produce a phytoalexin called ipomeamarone [6]. Ipomeamarone is a secondary metabolite of sesquiterpenes that acts as a toxic substance to domestic animals. Because the production of phytoalexins makes the sweetpotato bitter, astringent, and even more toxic to animals, the tuberous roots damaged by weevils are not fit for consumption by humans or livestock [5,6].

To develop resistant varieties, the identification of resistance genes and the elucidation of the mechanisms underlying weevil resistance in sweetpotato are necessary. Previous studies have reported the differences in weevil susceptibility among sweetpotato varieties [7,8,9]. Yada and colleagues performed genetic analysis on an F1 mapping population derived from the resistant African landrace New Kawago and susceptible North American cultivar Beauregard and identified simple sequence repeat markers associated with weevil resistance [10]. From the pest side, one group performed a transcriptome analysis on sweetpotato weevil (*Cylas puncticollis* Boheman) and discovered the presence of a functional RNAi pathway that may be used as a new strategy for controlling this pest [11]. Using next-generation sequencing (NGS) technology, Okada et al. performed genome-wide association studies (GWAS) in sweetpotato and detected several genomic regions associated with weevil resistance [12]. However, there are no reports regarding the molecular mechanisms underlying weevil resistance in sweetpotato using transcriptome-based analysis. Recently, with the decreasing costs and increasing throughput of NGS technology, several groups have reported large-scale transcriptome studies in sweetpotato, revealing the key genes and a comprehensive knowledge of the mechanisms underlying important agricultural traits [13,14,15,16,17,18,19,20]. In addition, the whole genome sequence and functionally annotated genes of the closely related diploid species *Ipomoea trifida* have been previously released [21,22,23]. Because *I. trifida* is considered a model sweetpotato species, its high-quality genome and gene sequences can be utilized as a reference for the transcriptome analysis of other sweetpotato varieties.

In this study, we performed an RNA sequencing (RNA-seq)-based transcriptome analysis using resistant and susceptible sweetpotato cultivars to comprehensively analyze the differentially expressed genes (DEGs) that respond to the feeding damage caused by weevils. Specifically, we aimed to identify the related genes and to elucidate the molecular mechanisms associated with weevil resistance in sweetpotato.

## 2. Results

### 2.1. Phenotyping for Weevil Resistance

**Figure 1:**
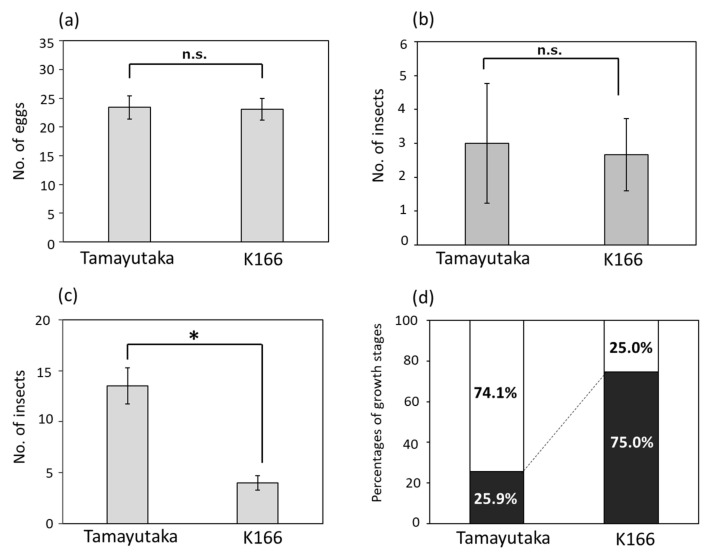
Results of the weevil resistance tests between the susceptible Tamayutaka and resistant Kyushu No. 166 (K166) cultivars. (**a**) The average number of eggs per tuberous root at 3 days after inoculation; (**b**) the average number of insects per tuberous root at 15 days after inoculation; (**c**) the average number of insects at 30 days after inoculation; (**d**) the growth state of insects at 30 days after inoculation. Black and white bars indicate the ratios of the larvae and pupae, respectively. * *p* < 0.05, n.s.: not significant.

In the weevil resistance evaluation test, 10 adult West Indian sweetpotato weevils (*Euscepes postfasciatus* (Fairmaire)) (sex ratio, male/female = 1:1) were inoculated into the sweetpotato tubers of weevil-resistant Kyushu No. 166 (K166) and susceptible Tamayutaka cultivars. The degree of damage was investigated by counting the number of eggs on the surface of tuberous roots at 3 days after inoculation and the total number of insects at 15 and 30 days after inoculation. In addition, the growth stages of the insects were investigated at 30 days after inoculation. At 3 days after inoculation, the average number of eggs per tuberous root was 23.1 and 23.4 for K166 and Tamayutaka, respectively, and no significant difference was detected between the two (Figure 1a). At 15 days after inoculation, the average number of insects per tuberous root was 2.7 and 3.0 for K166 and Tamayutaka, respectively, and again no significant difference was observed (Figure 1b). In contrast, the average number of insects at 30 days after inoculation was 4.0 and 13.5 for K166 and Tamayutaka, respectively, with a significant difference (*p* < 0.05) in the total number of insects between the two cultivars (Figure 1c). In K166, the total numbers of insects were six larvae (75.0%) and seven pupae (25.0%). On the other hand, of the total number of insects in Tamayutaka, the numbers of larvae and pupae were 7 (25.9%) and 20 (74.1%), respectively (Figure 1d). Thus, at 30 days after inoculation, there was a large difference in the number and growth state of insects between the two cultivars. These results suggest that weevils have no preferred spawning sites between the two cultivars, and that the resistant K166 suppressed weevil growth, especially during pupation.

### 2.2. RNA-Seq-Based Transcriptome Analysis

**Figure 2:**
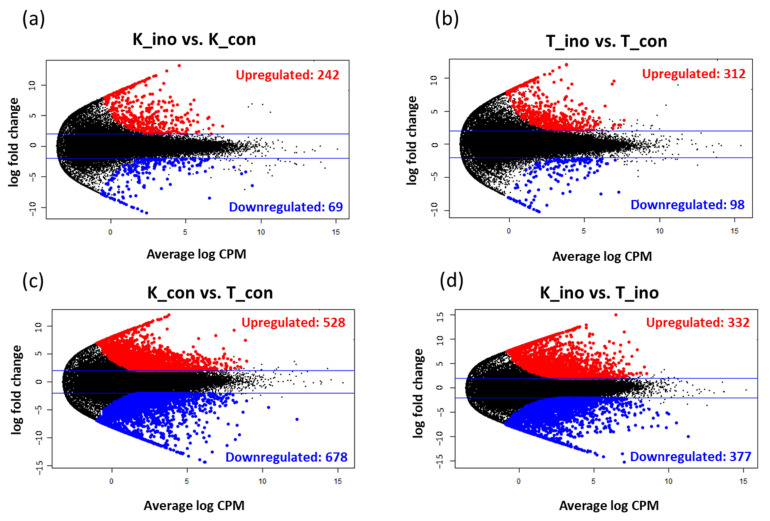
MA plots of the identified differentially expressed genes (DEGs). Red and blue dots represent the upregulated and downregulated DEGs, respectively. Black dots represent the non-differentially expressed genes. (**a**) DEGs between the control (K_con) and inoculation (K_ino) plots of Kyushu No. 166 (K166); (**b**) DEGs between control (T_con) and inoculation (T_ino) plots of Tamayutaka; (**c**) DEGs between the control plots of K166 and Tamayutaka; (**d**) DEGs between the inoculation plots of K166 and Tamayutaka.

RNA-seq analysis was performed to identify the DEGs between the weevil-resistant and susceptible cultivars. Total RNA was extracted for RNA-seq library preparation using tuberous roots from two experimental plots (control and inoculation) with two replicates. Sequencing with HiSeqX produced a total of 219,712,549 reads for all samples (22,546,509–43,206,139). After preprocessing, a total of 209,834,637 reads (21,472,899–41,409,345) were obtained (Table S1). The average alignment rate was 72.79% (61.64–78.73%) after mapping the reads to the transcriptome sequences of *I. trifida* (Table S2). Using eXpress and edgeR, differential expression analysis between the weevil inoculation and control plots revealed 242 upregulated and 69 downregulated DEGs in K166 and 312 upregulated and 98 downregulated DEGs in Tamayutaka (Figure 2a,b, Supplementary Tables S3–S7), confirming the transcriptional response to the damage by weevil feeding in both cultivars. In contrast, 528 upregulated and 678 downregulated DEGs were detected between the control plots of K166 and Tamayutaka, indicating that there are many DEGs between the two cultivars (Figure 2c, Tables S3, S8 and S9). From the results of 1206 DEGs detected between cultivars, even in the control plot, the genetic backgrounds of these two cultivars were considered to be quite different. Moreover, 332 upregulated and 377 downregulated DEGs were detected between the weevil inoculation plots of K166 and Tamayutaka (Figure 2d, Tables S3, S10 and S11).

**Figure 3:**
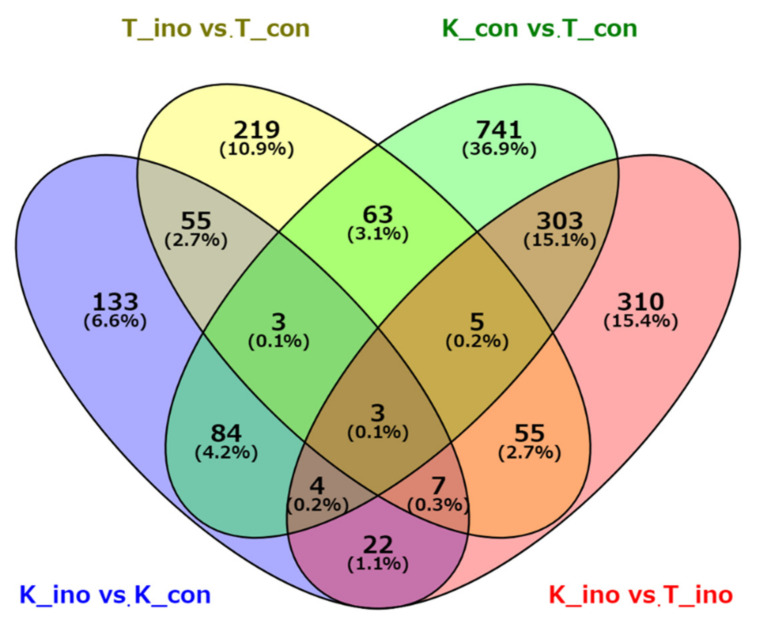
Venn diagram showing the number of DEGs and the overlaps among the four comparisons.

In addition, we investigated the number of DEGs across four comparisons (K_ino vs. K_con, T_ino vs. T_con, K_con vs. T_con, and K_ino vs. T_ino) and the overlaps between each set of DEGs. Venn diagram analysis indicated that a total of 68 (55 + 3 + 3 + 7) DEGs were differentially expressed in both T_ino vs. T_con and K_ino vs. K_con conditions (Figure 3), suggesting that the expression levels of a relatively small number of these genes changed commonly in both varieties in response to weevil feeding damage. On the other hand, a total of 315 (303 + 5 + 3 + 4) DEGs were commonly detected between K_con vs. T_con and K_ino vs. T_ino comparisons, suggesting that these hundreds of DEGs showed different expression levels between cultivars regardless of control or inoculation plots. Interestingly, a total of 741 (36.9%) and 310 (15.4%) DEGs were detected that were present only in the control (comparison of K_con vs. T_con) and inoculation plots (comparison of K_ino vs. T_ino), indicating that there are many genes with expression levels that changed specifically in each experimental plot.

**Figure 4:**
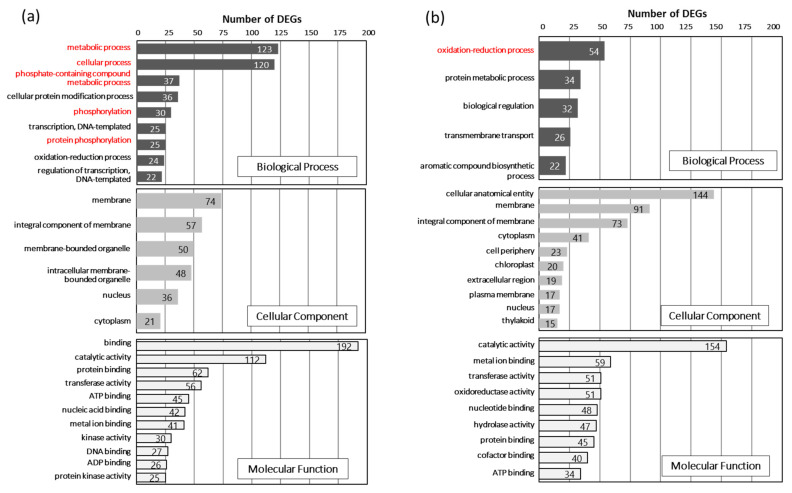
The enriched gene ontology (GO) terms of the identified differentially expressed genes (DEGs) between Kyushu No. 166 (K166) and Tamayutaka at 30 days after inoculation. The GO terms were classified in three categories: biological process, cellular component, and molecular function. (**a**) The top GO terms for the upregulated DEGs in K166; (**b**) the top GO terms for the upregulated DEGs in Tamayutaka.

Gene ontology (GO) analysis was performed using OmicsBox for the functional annotation of the identified DEGs between K166 and Tamayutaka at 30 days after inoculation. In the biological process category, the top two enriched GO terms for the set of genes upregulated in K166 were “metabolic process” and “cellular process”, followed by terms related to phosphorylation such as “phosphate-containing compound metabolic process”, “phosphorylation”, and “protein phosphorylation” (Figure 4a). In terms of molecular function, “binding” and “catalytic activity” were prominently represented. On the other hand, the top enriched GO terms for the upregulated genes in Tamayutaka were “oxidation-reduction process”, “cellular anatomical entity”, and “catalytic activity” in the biological process, cellular component, and molecular function categories, respectively (Figure 4b). In the control plot, the GO terms related to phosphorylation, metabolic, and cellular processes were not enriched in the upregulated DEGs of K166 (Figure S1). These results suggest that the upregulation of the genes involved in phosphorylation, metabolic, and cellular processes contributes to weevil resistance in K166.

### 2.3. Juvenile Hormone (JH)-Related Genes

**Figure 5:**
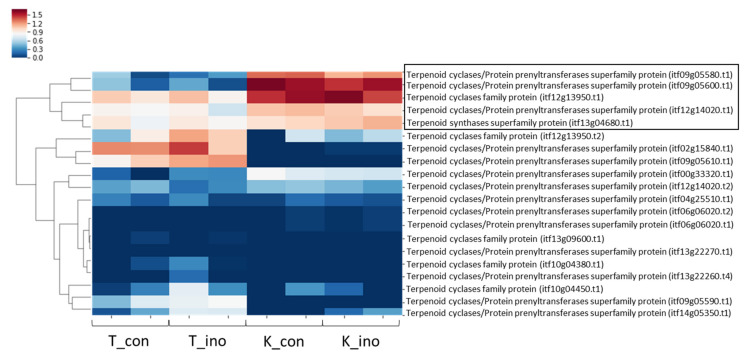
Hierarchical clustering and expression heatmap of 20 terpenoid-related genes. Black boxes indicate the genes that are highly differentially expressed between Kyushu No. 166 (K166) and Tamayutaka. The gene expression level (log2 fold change value) is represented by the blue (low) to red (high) color gradient. K_con: K166 samples in the control plot, K_ino: K166 samples in the inoculation plot, T_con: Tamayutaka samples in the control plot, T_ino: Tamayutaka samples in the inoculation plot.

Based on the results of the weevil inoculation test, we hypothesized that the resistance trait of K166 was likely to be due to the suppression of pupation during larval development. Hence, we analyzed the JH-related genes involved in the suppression of insect metamorphosis. Larval–pupal and pupal–adult transitions are controlled by the action of JHs and molting hormones in insects such as silkworms (*Bombyx mori* L.). JHs and JH analogues (JHAs or juvenoids) are known to prolong larval life by inhibiting the larval–pupal and pupal–adult transitions [24]. This mechanism is conserved in many insect species. In contrast, terpenes are a large and diverse class of organic compounds produced by a variety of plants. The biochemical actions of natural insect JHs and plant terpenes and terpenoid compounds are similar because terpenes mimic the action of insect JHs [24]. Therefore, it is possible that weevils may mistakenly recognize the JHs produced by sweetpotato, which may explain the suppression of weevil pupation in K166. To verify this hypothesis, we also investigated the expression levels of terpenoid-related genes and discovered 20 genes that were present in K166 and Tamayutaka (Figure 5). Five genes (*itf09g05600.t1*, *itf12g13950.t1*, *itf09g05580.t1*, *itf13g04680.t1*, and *itf12g14020.t1*) were highly expressed in K166 (Figure 5). Of these five genes, three (*itf09g05600.t1*, *itf09g05580.t1*, and *itf12g13950.t1*) had significantly increased expression in K166 (Figure S2, Tables S8 and S10), suggesting that these may be candidate genes that contribute to the inhibition of weevil pupation in K166. Interestingly, two (*itf09g05600.t1* and *itf09g05580.t1*) were found to be very closely located on chromosome 9, with a physical distance of approximately 2 kilobase (kb) from each other. The amino acid sequences of the two genes are also highly conserved (Figure S3). In addition, two functional domains (N-terminal and metal-binding) related to terpene synthase were present in both genes (Figure S4), suggesting their potential roles in terpene synthesis.

In addition, we focused on the disease resistance-related genes and investigated their expression levels in both cultivars. By searching for genes that had resistance-related annotations and were highly expressed in the resistant K166 cultivar after inoculation, 30 genes were detected (Figure S5). There are many genes with annotations such as “NB-ARC domain-containing disease resistance protein”, “TIR-NBS-LRR class”, and “CC-NBS-LRR class”. On the contrary, only nine genes were detected with resistance-related annotations and higher expression levels in Tamayutaka (data not shown). These results indicate that more disease-resistance genes were highly expressed in the resistant cultivar, which may contribute to resistance to some extent.

## 3. Discussion

In this study, we performed a comprehensive transcriptome analysis of weevil resistance in sweetpotato. Although weevils are considered a serious pest worldwide, this insect species is distributed in specific regions only, such as the Nansei Islands in Japan. However, due to global warming, the distribution of weevils is expected to expand further, and weevil resistance may become the most desirable agricultural trait for sweetpotato cultivation in the future. To date, there have been no reports of NGS-based comprehensive gene expression analysis focused on investigating weevil resistance in sweetpotato. Therefore, our study provides novel insights into the transcripts that respond to damage by weevil feeding in sweetpotato.

RNA-seq analysis of weevil-resistant and susceptible cultivars revealed numerous DEGs between the two. Even in the control plots, many DEGs (1206) were detected between the two cultivars, reflecting the difference in their genetic backgrounds. Based on the pedigree information (Figure S6), the genetic backgrounds of the two cultivars are considered to be quite different. There were more upregulated than downregulated DEGs after weevil inoculation (98 downregulated and 312 upregulated DEGs in Tamayutaka, 69 downregulated and 242 upregulated DEGs in K166), indicating that transcriptional responses to the feeding damage by weevils occurred in both cultivars. Functional analysis of the DEGs also revealed that after weevil inoculation, the transcription of genes associated with metabolic processes, cellular processes, and phosphorylation were upregulated in the resistant K166 cultivar, suggesting that these genes may be critical for weevil resistance. On the other hand, it should be noted that while these DEGs may have contributed to weevil resistance, their expression levels may be the result of a response to feeding damage by weevils.

Plants possess a complex defense system against diverse pests and pathogens and a response system composed of pathogen detection, signal transduction, and defense response [25]. Plants can perceive certain elicitors in insect oral secretions that enter wounds during feeding and rapidly activate mitogen-activated protein kinase (MAPK) signaling [26]. MAPKs play critical roles in plant resistance against insect herbivores by regulating the herbivory-induced changes in phytohormones, the transcriptome activation of herbivore defense-related genes, and the accumulation of defensive metabolites. MAPKs consist of 11 domains that are found in all serine/threonine protein kinases [27], which are activated by the dual phosphorylation of the Thr and Tyr residues in the TxY motif of the activation loop (T-loop) located between subdomains VII and VIII. In the T-loop, activation occurs via MAPK kinases (MAPKKs), which are activated by MAPKK kinases (MAPKKKs) through the phosphorylation of conserved Ser and/or Thr residues. Activated MAPKs phosphorylate their substrates, including the transcription factors and enzymes that trigger downstream stress-related responses [26]. Thus, MAPK activation via phosphorylation may have immediately occurred after wounding and feeding by weevil larvae in K166, which subsequently induced defense reactions via phosphorylation of associated transcription factors and proteins.

In contrast, the expression of genes related to the oxidation-reduction process and oxidoreductase activity were upregulated in the susceptible Tamayutaka cultivar. Sweetpotato contains several phytoalexins, collectively known as furanoterpenoids, such as ipomeamarone and its precursor dehydroipomeamarone, ipomeanine, 1-ipomeanol, 4-ipomeanol, and 1,4-ipomeadiol [28,29,30]. Ipomeanine is produced by the oxidation of 4-ipomeanol, whereas 1,4-ipomeadiol and ipomeanol are produced by the reduction of ipomeanine [31]. After weevil inoculation, Tamayutaka was found to be more damaged than K166; thus, the production of phytoalexins such as ipomeamarone and ipomeanine was expected to be high in response to the damage. Consequently, a higher number of upregulated DEGs related to the oxidation-reduction process were detected in Tamayutaka than in K166. Furthermore, the upregulation of these genes was observed in the inoculation plots only (Figure S7), suggesting that both cultivars were damaged by weevil feeding and the resulting oxidation-reduction processes.

In the weevil inoculation test, there was no significant difference in the number of eggs at 3 days after inoculation or the total number of insects at 15 days after inoculation between the resistant and susceptible cultivars. In contrast, there was a large difference in the total number of insects and the growth stage at 30 days after inoculation. These results suggest that weevils have no preferred spawning sites between resistant and susceptible cultivars. However, the weevil growth, particularly pupation, was suppressed in K166, indicating that one possible mechanism of weevil resistance is the inhibition of weevil development and reproduction. We tested this hypothesis by investigating the JHs involved in the suppression of insect metamorphosis. A Krüppel homolog 1 gene (*Kr-h1*), which is induced by the JH via a JH receptor, plays a key role in the repression of insect metamorphosis [32]. The transcription factor Broad-Complex (*BR-C*) functions as a “pupal specifier” in the larval–pupal transition; JH-inducible Kr-h1 binds to the *BR-C* promoter region and represses its transcription, resulting in the inhibition of larval–pupal transition [33,34]. Therefore, JHs can inhibit larval–pupal and pupal–adult transitions in holometabolous insects. In such cases, the larva reaches the end of its life, and the reproduction of the next generation is halted. On the other hand, plant-derived metabolites are known to act as JHAs in insects, and the biochemical actions of insect JHs and plant terpenes and terpenoid compounds are similar [30]. In K166, the weevil may have misrecognized the JH produced by sweetpotato, resulting in the suppressed pupation of the larvae. Therefore, we also investigated the expression levels of terpenoid-related genes and discovered five genes that were upregulated in K166. In particular, three of the five genes had significantly increased expression levels in K166. Among the three genes, two possessed the N-terminal and metal-binding domains of terpene synthase, suggesting that the two genes may function in terpene synthesis and may be associated with the inhibited pupation of weevil larvae in K166. Interestingly, resistant sweetpotato cultivars may possess weevil-recognizing plant compounds that cause growth retardation in weevils. In addition, we revealed that more disease resistance-related genes were highly expressed in the resistant cultivar. These genes may also contribute to the expression of resistance in K166.

Therefore, future studies should investigate whether there is a difference in the amount of terpenes produced by weevil-resistant and susceptible cultivars and determine the correlation between the expression of terpenoid-related genes and the amount of terpenes produced. Furthermore, we are planning to perform additional genetic analyses such as quantitative trait loci (QTL) mapping and GWAS using the F1 populations derived from K166 and Tamayutaka. By investigating the DEGs in the selected QTL region, we can identify the candidate genes controlling weevil resistance in K166.

## 4. Materials and Methods

### 4.1. Resistance Evaluation Test by Weevil Inoculation

The weevil-resistant K166 and susceptible Tamayutaka cultivars were chosen for this study. K166 is the progeny of a cross between Kyukei98160-1 and Murasakimasari. Tamayutaka is derived from a cross between Kanto No. 33 and Kuroshirazu (Figure S6). The breeding process and pedigree information are shown in Figure S6. For all plant samples, the tuberous roots produced in 2017 and cultivated at the National Agrobiological and Food Research Organization for Kyushu Okinawa Region (Miyakonojo City, Miyazaki Prefecture, Japan) were used. The weevil inoculation test and resistance evaluation were conducted at the Kyushu Okinawa Agricultural Research Center (Itoman City, Okinawa Prefecture, Japan). Adult West Indian sweetpotato weevils (*Euscepes postfasciatus* (Fairmaire)) were used for the inoculation test, and each sweetpotato sample was placed in a plastic case. We prepared two experimental plots (control and inoculation) with three biological replicates. In the inoculation plot, one sweetpotato tuber root was inoculated with 10 adults (sex ratio, male/female = 1:1). The degree of damage to the tuberous roots was investigated at 3, 15, and 30 days after inoculation. At 3 days after inoculation, the number of eggs on the surface of the tuberous root was counted. At 15 and 30 days after inoculation, the sweetpotato samples were dissected, and the degree of damage was investigated by counting the number of larvae, pupae, and adults in the tuberous roots.

### 4.2. RNA Extraction, Library Preparation, and RNA-Seq

The tuberous roots from the control and inoculation groups with two replicates were collected. Total RNA was extracted using the phenol–chloroform method. Lithium chloride (LiCl) precipitation was performed to remove any impurities, and the extracted RNA was purified using RNeasy Plant Mini Kit (Qiagen, Hilden, Germany). The RNA yield (ng/µL) was measured using Qubit fluorometer (Thermo Fisher Scientific, Waltham, MA, USA), whereas the RNA quality was confirmed by agarose gel electrophoresis. The RNA-seq library was prepared using the KAPA mRNA HyperPrep Kit (KAPA Biosystems, Woburn, MA, USA). The library concentration of each sample was also measured using Qubit (Thermo Fisher Scientific). All samples were pooled in equal volumes to prepare the libraries for RNA-seq, and sequencing with HiSeqX (Illumina, San Diego, CA, USA) yielded 150-base pair (bp) paired-end reads.

### 4.3. Data Analysis

The obtained RNA-seq data were analyzed using the following procedures. First, the quality of the paired-end reads was determined using FastQC [35]. Second, the adapter sequences and low-quality nucleotides were removed using Cutadapt [36]. The threshold value of the quality score was 30, and the minimum read length for trimming was 50 bp. After preprocessing, the reads were checked again by FastQC to confirm the quality. Third, using the transcriptome sequence of the publicly available sweetpotato diploid wild species *I. trifida* [22] as the reference, the preprocessed reads were aligned using Bowtie2 software [37]. Fourth, the gene expression levels were determined using eXpress (https://pachterlab.github.io/eXpress/index.html, accessed on 24 June 2019), and the DEGs were analyzed using edgeR [38]. Fifth, DEGs with false-discovery rate values < 0.05 and |log2 fold change| values > 2 were extracted for subsequent GO and enrichment analyses. Sixth, an in-house Python script was used to create a heatmap of the DEGs. Briefly, the transcripts per kilobase million (TPM) values from eXpress analysis were averaged within the iteration and then logarithmically converted and normalized to create a heatmap. The Venn diagram was generated using the online tool VENNY (v2.1) [39].

For the GO analysis, the FASTA sequences of the DEGs were imported into OmicsBox version 1.2 (BioBam) and aligned to the NCBI Viridiplantae NR database using blastx search (E-value ≤ 1.0 × 10^−3^). Subsequent GO mapping was performed using the Blast2GO mapping against the latest version of the GO database to obtain the functional labels [40,41]. Then, the appropriate GO term was assigned to predict the function of the annotated sequences using an e-value cutoff of 1.0 × 10^−6^ and an annotation cutoff of 55. Bar plots of the enriched GO terms were created for three categories: biological process, cellular component, and molecular function. For the candidate genes involved in weevil resistance, the homology of their amino acid sequences was confirmed using BioEdit. In addition, the functional gene domains were searched against several protein databases, including ProDom (http://prodom.prabi.fr, accessed on 5 November 2019) [42], Pfam (https://pfam.xfam.org, accessed on 5 November 2019) [43], SMART (http://smart.embl-heidelberg.de, accessed on 5 November 2019) [44], and PANTHER (http://pantherdb.org/, accessed on 5 November 2019) [45], using InterProScan [46].

## Data Availability

The RNA sequencing datasets were submitted to the DDBJ database. The accession number is DRA012193.

## Supplementary Materials

The following are available online at https://www.mdpi.com/article/10.3390/plants10081535/s1, Figure S1: The top GO terms for the upregulated DEGs in K166 in the control plot, Figure S2: The gene expression levels of the two candidate genes (*itf09g05600.t1* and *itf09g05580.t1*) based on the TPM values, Figure S3: The sequence homology of the amino acid sequences of the two genes (*itf09g05600.t1* and *itf09g05580.t1*), Figure S4: The gene structure and functional domains of the two genes, Figure S5: Hierarchical clustering and expression heatmap of 30 disease resistance-related genes, Figure S6: The breeding process and pedigree information of K166 and Tamayutaka, Figure S7: The enriched GO terms of the identified DEGs between the weevil inoculation and control plots, Table S1: The number of raw and preprocessed reads obtained through RNA sequencing, Table S2: Alignment rate of RNA sequencing reads, Table S3: Summary of the identified DEGs, Table S4: Upregulated genes between the weevil inoculation and control plots of Kyushu No. 166 (K166), Table S5: Upregulated genes between the weevil inoculation and control plots of Tamayutaka, Table S6: Downregulated genes between the weevil inoculation and control plots of Kyushu No. 166 (K166), Table S7: Downregulated genes between the weevil inoculation and control plots of Tamayutaka, Table S8: Upregulated genes between the control plots of Kyushu No. 166 (K166) and Tamayutaka, Table S9: Downregulated genes between the control plots of Kyushu No. 166 (K166) and Tamayutaka, Table S10: Upregulated genes between the weevil inoculation plots of Kyushu No. 166 (K166) and Tamayutaka, Table S11: Downregulated genes between the weevil inoculation plots of Kyushu No. 166 (K166) and Tamayutaka.
