# Supplementary material for: Transcriptome Analysis Reveals Key Genes Involved in Weevil Resistance in the Hexaploid Sweetpotato
Source: Plants (Basel). 2021 Jul 27;10(8):1535. doi: 10.3390/plants10081535 (PMC8398197; doi:10.3390/plants10081535)
Supplement: Supplementary file 1 [file plants-10-01535-s001.zip › Figure_S1.pdf]

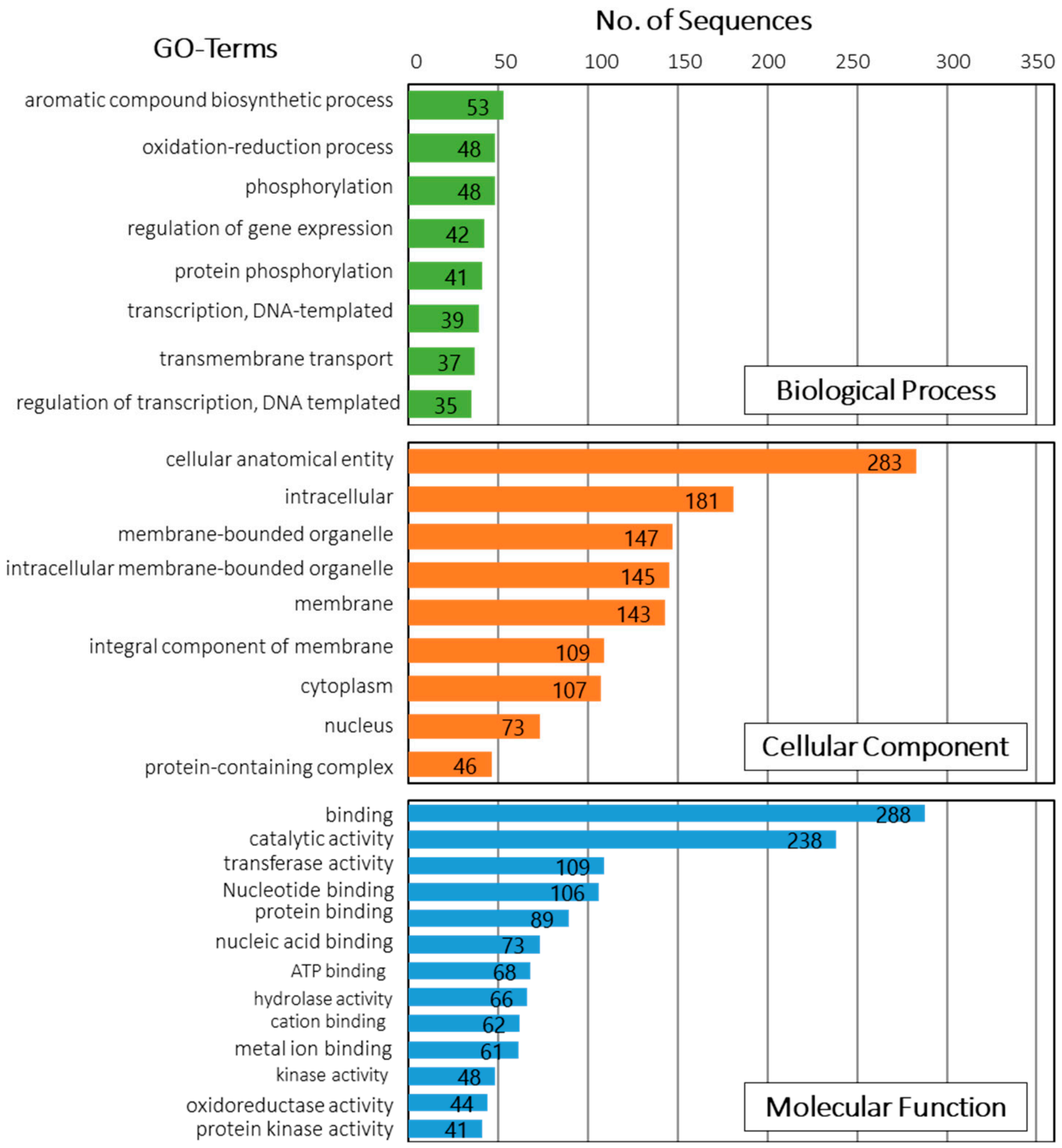

Supplemental Figure 1. The enriched GO terms of the identified DEGs between K166 and Tamayutaka in the control plot. Here, the top GO terms for the upregulated DEGs in K166 are shown.
