# Supplementary material for: Transcriptome Analysis Reveals Key Genes Involved in Weevil Resistance in the Hexaploid Sweetpotato
Source: Plants (Basel). 2021 Jul 27;10(8):1535. doi: 10.3390/plants10081535 (PMC8398197; doi:10.3390/plants10081535)
Supplement: Supplementary file 1 [file plants-10-01535-s001.zip › Figure_S2.pdf]

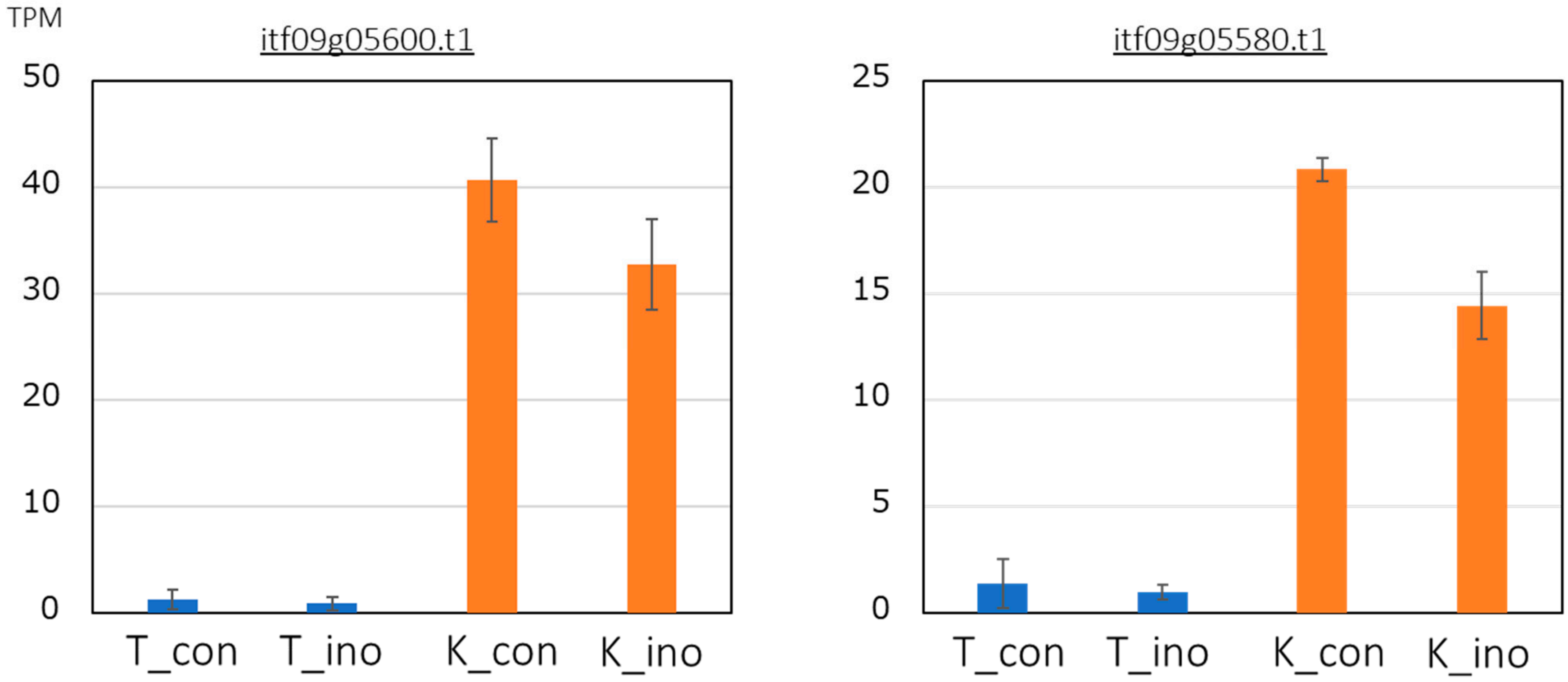

Supplemental Figure 2. Gene expression level of the candidate two genes (itf09g05600.t1, itf09g05580.t1) based on the TPM values.
