# Supplementary material for: Transcriptome Analysis Reveals Key Genes Involved in Weevil Resistance in the Hexaploid Sweetpotato
Source: Plants (Basel). 2021 Jul 27;10(8):1535. doi: 10.3390/plants10081535 (PMC8398197; doi:10.3390/plants10081535)
Supplement: Supplementary file 1 [file plants-10-01535-s001.zip › Figure_S3.pdf]

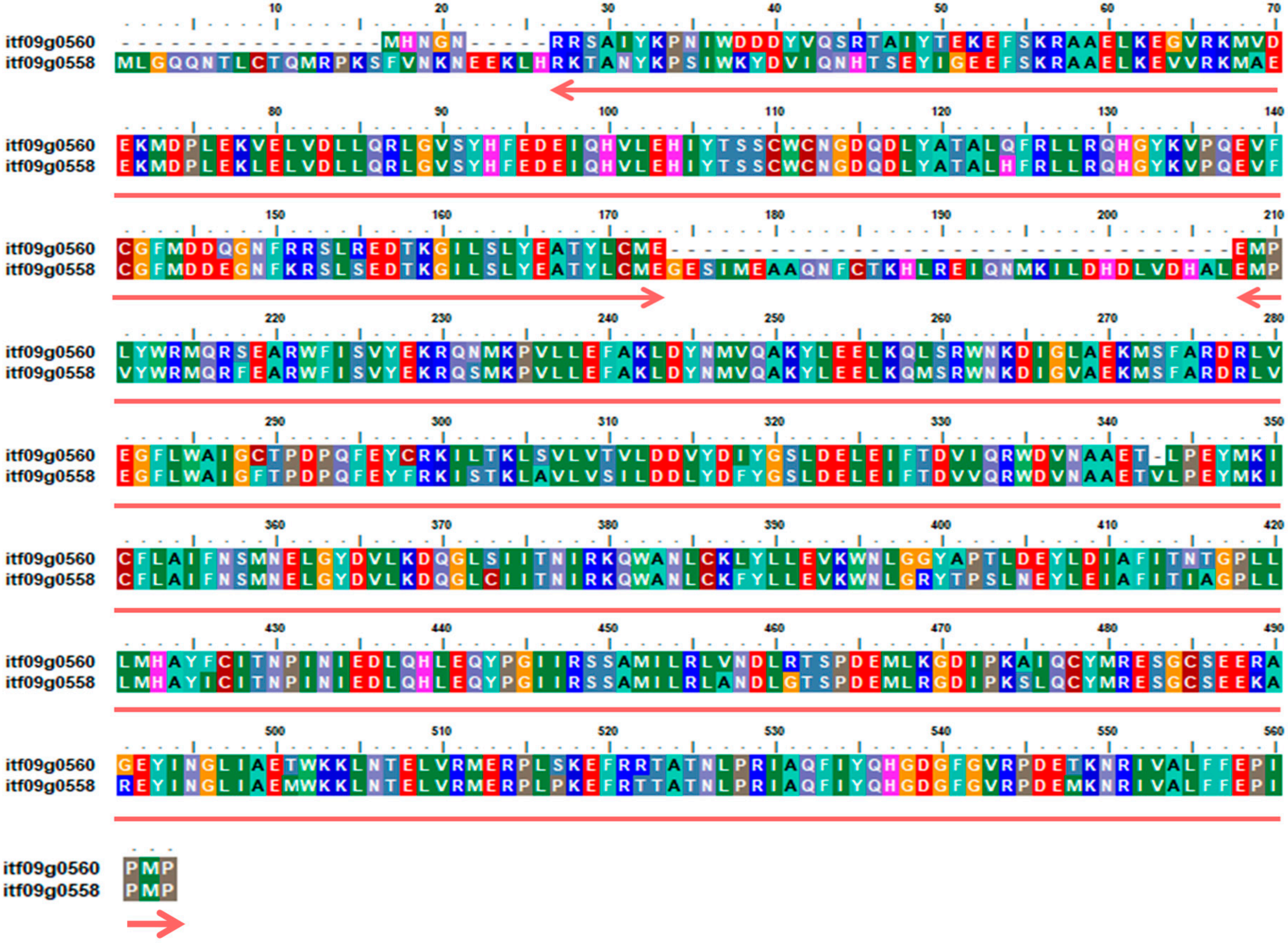

Supplemental Figure 3. The sequence homology of the amino acid sequences of the two genes (itf09g05600.t1, itf09g05580.t1). Red arrow indicates the conserved region between the two genes.
