# Supplementary material for: Transcriptome Analysis Reveals Key Genes Involved in Weevil Resistance in the Hexaploid Sweetpotato
Source: Plants (Basel). 2021 Jul 27;10(8):1535. doi: 10.3390/plants10081535 (PMC8398197; doi:10.3390/plants10081535)
Supplement: Supplementary file 1 [file plants-10-01535-s001.zip › Figure_S5.pdf]

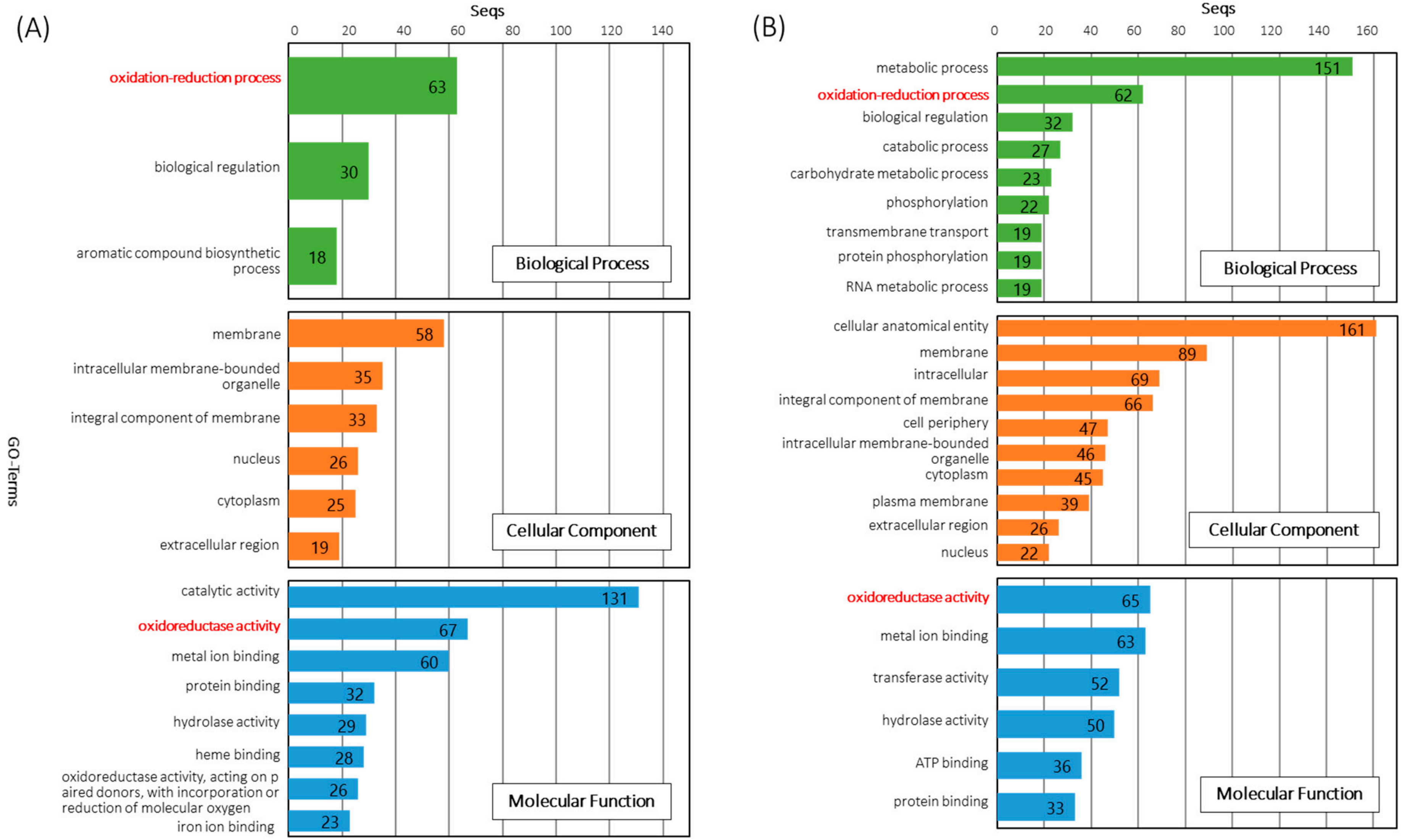

Supplemental Figure 5. The enriched GO terms of the identified DEGs between the weevil inoculation and control plots. (A) The top GO terms for the upregulated DEGs in the inoculation plot of K166. (B) The top GO terms for the upregulated DEGs in the inoculation plot of Tamayutaka. GO terms with red color indicated ‘oxidation-reduction process’ and ‘oxidoreductase activity’.
