# Supplementary figures and images for: Transcriptome Analysis Reveals Key Genes Involved in Weevil Resistance in the Hexaploid Sweetpotato
Source: Plants (Basel). 2021 Jul 27;10(8):1535. doi: 10.3390/plants10081535 (PMC8398197; doi:10.3390/plants10081535)

**itf09g05600.t1**

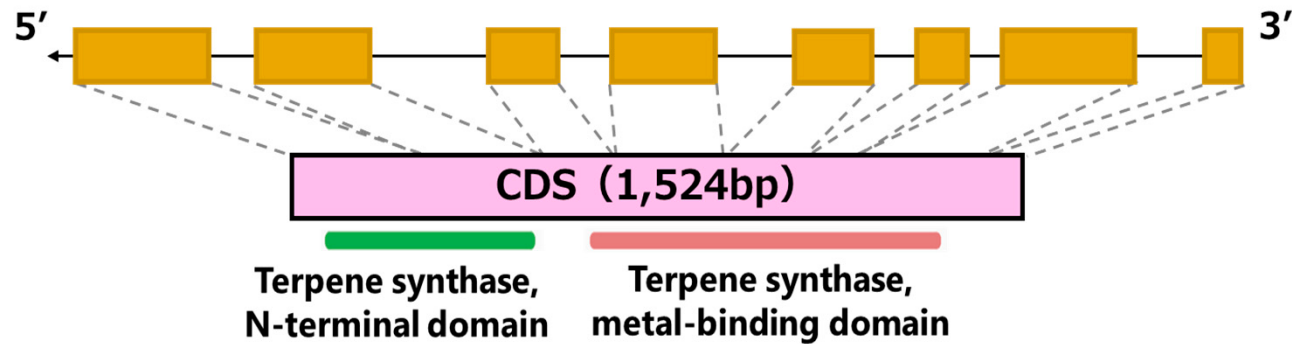

**itf09g05580.t1**

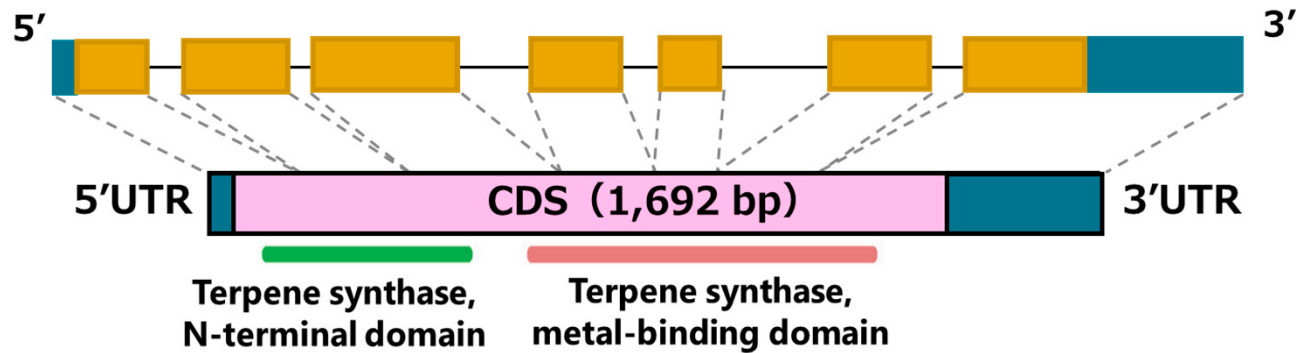

Supplemental Figure 4. Gene structure and functional domains of the two genes.

Supplement: Supplementary file 1 [file plants-10-01535-s001.zip › Figure_S4.pdf]
